# Estimating a Markovian Epidemic Model Using Household Serial Interval Data from the Early Phase of an Epidemic

**DOI:** 10.1371/journal.pone.0073420

**Published:** 2013-08-30

**Authors:** Andrew J. Black, Joshua V. Ross

**Affiliations:** 1 School of Mathematical Sciences, The University of Adelaide, Adelaide, Australia; Northeastern University, United States of America

## Abstract

The *clinical serial interval* of an infectious disease is the time between date of symptom onset in an index case and the date of symptom onset in one of its secondary cases. It is a quantity which is commonly collected during a pandemic and is of fundamental importance to public health policy and mathematical modelling. In this paper we present a novel method for calculating the serial interval distribution for a Markovian model of household transmission dynamics. This allows the use of Bayesian MCMC methods, with explicit evaluation of the likelihood, to fit to serial interval data and infer parameters of the underlying model. We use simulated and real data to verify the accuracy of our methodology and illustrate the importance of accounting for household size. The output of our approach can be used to produce posterior distributions of population level epidemic characteristics.

## Introduction

A quantity which is commonly recorded during a pandemic is the *clinical serial interval*, defined as the time between date of symptom onset in an index case and the date of symptom onset in one of its secondary cases [1]–[3]. It was one of the main quantities recorded, at the level of households, during the 2009 H1N1 pandemic and subsequently used for understanding the dynamics of the pandemic [1], [3]–[6]. Numerous studies have illuminated the critical dependence of disease dynamics and choice of control policy on this quantity through its relation to the generation time [7]–[10].

A common and simple way to analyse serial interval data is to fit it with a parametric distribution [4], [11]–[13]. This approach allows an accurate calculation of the mean and possibly other moments. However, an obvious drawback of such an approach is that the estimate itself gives no understanding of the underlying mechanics, and hence it is difficult to make predictions with quantifiable confidence or to assess the impact of proposed control policies. This is because the serial interval is not a biological quantity in its own right but the convolution of the processes of transmission and incubation. This is further confounded by the fact that the time of infection is almost certainly unobservable, and because households are small, depletion of susceptibles has a large impact on the (stochastic) transmission process [14]. For these reasons, the only way to infer both epidemiological and dynamical quantities from serial interval data is by assuming and fitting a transmission model [15], [16]. This approach not only provides an estimate of the serial interval distribution, but estimates a full mechanistic model which may be used to make predictions and assess the impact of intervention policies [6].

A type of transmission model which has been growing in popularity, especially when considering household structure, are Markovian models [6], [17]–[20]. In these it is assumed that there are two levels of mixing: strong mixing within a household and weaker mixing between households [17]. As the overall population is assumed to be large and randomly mixing, then during the early stages of an epidemic repeated introduction of infection into a single household is negligible. The assumption of only one introduction allows for deeper analysis of the model, and also allows for computationally-efficient methods to be developed for evaluating early-time quantities [17], [21]; here it allows us to ignore the external infection rate, and use serial interval data to estimate the other parameters. Obviously during the mid-to-late stages of an outbreak, this assumption breaks down and hence more complex models are required, but for this study we confine ourselves to this common assumption. This early stage of an epidemic is important as we want to infer parameters which can then be used (with further assumptions) in population level models to assess possible interventions and inform public health policy.

In this paper we show how to fit a quite general Markovian household epidemic model using serial interval data. This is achieved by first explaining how the serial interval distribution can be calculated for this model, and hence used to derive exact likelihoods. We then use this for parameter inference via Bayesian Markov chain Monte Carlo (MCMC) methods. We investigate the accuracy of this methodology via simulation studies and illustrate its use with data previously studied from a household transmission study of seasonal influenza in Hong Kong [13]. Our investigations identify that household size has an appreciable impact on the serial interval distribution and that incorporating household size data into inference methods allows more accurate estimates of model parameters.

The advantages of our methodology are threefold: Firstly, it is fully stochastic and mechanistic – the former is vital given the average size of a household and the latter leads to greater understanding of the epidemic. Secondly, we can numerically solve the model, and hence calculate the serial interval distribution exactly to an arbitrary precision – there is no need for approximations by branching processes or for assumptions of independence in order to derive results. Thirdly, it is very computationally efficient. This means we can achieve the methodological ideal of full evaluation of the uncertainty in parameter estimates and derive accurate credible intervals for all results. Efficiency also allows for the potential inclusion of much more epidemiological detail in future models were more data available in such studies.

## Methods

We assume a continuous time Markovian model for the dynamics of a disease within a household of size N. We use a general 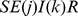 model, where the exposed and infectious periods are split up into 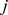 and 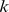 phases so that each has an Erlang distribution with mean exposed and infectious periods 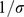 and 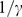, and variances 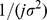 and 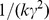, respectively [22], [23]. Infection is assumed to be frequency dependent (but density-dependent transmission is no obstacle to the methodology we outline, and will be discussed later) with transmission parameter 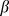 [15], [24]. The transition rates for this model are given in Table 1.

**Table 1:** Within household dynamics.

Event	Transition	Rate
Infection	(*S, E_1_*) →(*S −1,E_ 1_+1*)	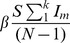
Exposed progression,	(*E_n_,E_n+1_*) →(*E_n_−1,E_n+1_+1*)	*j*σ*E_n_*
(*n = 1*,…,*j−1*)		
Start shedding	(*E_j_,I_1_*) →( *E_j_−1,Ι_1_+1)*	*j*σ*E_j_*
Infection progression,	(*I_m,_I_m+1_*) →(*Ι_m_−1,Ι_m+1_+1*)	*k γI_m_*
(*m = 1*,…,*k−1*)		
Recovery	*I_k_* →*Ι_k_−1*	*k γI_k_*

The transitions and associated rates which define the stochastic *SE(j)I(k)R* model for the within-household dynamics.

The model is specified by the matrix 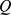, which encodes the transition rates between different possible states of the household [6], [18]. For the 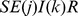 model we consider, the total number of possible states is
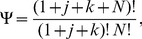
(1)


hence this is also the dimension of 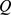. The element 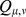 is the rate of transition from state 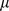 to 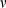 for 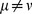, where 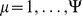 and 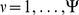. 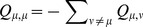, is the negative of the total rate at which the system leaves state 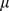. The dynamics of the model are then given by the equation,
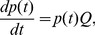
(2)where 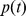 is the probability vector with 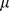 th entry the probability of the household being in state 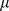 at time 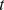 [18]. As we are dealing with household models, the dimension of 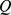, given by Equation (1), is relatively small, so Equation (2) can be solved efficiently using matrix exponential methods [25]. Hence we can calculate 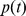 to an arbitrary precision, side-stepping the need for any type of potentially costly simulation.

To calculate the serial interval distribution we need to evaluate the probability of observing a secondary case in a given time interval, given that we start with an index case at time 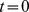. To do this we first form the transition rate matrix 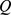 corresponding to the 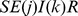 model for a given household size and set of parameters.

We assume that the appearance of symptoms coincides with entering the (first) infectious class [26]; in the later sections we discuss how Markovian models can be extended to weaken this assumption. The 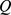 matrix is then modified so that states which correspond to a serial interval event – a second individual entering the first infectious class – are made absorbing. If we order the states of the system by 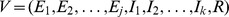, then the set of absorbing states are
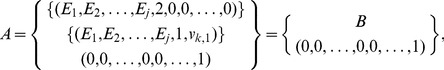
(3)where 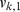 is the set of all vectors of length 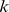, with a 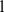 in a single position and zeros elsewhere. The set of states 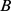 are those corresponding to serial interval events, while the last one is recovery with no further infection. This model explicitly takes into account that the second person to start showing symptoms might not have been the first to be infected, and hence evaluates the probabilities associated with the clinical serial interval.

For a household of size 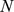, the initial state of the chain is set as 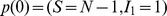. In doing this we are implicitly assuming that the first person to show symptoms is also the first person to introduce infection into the household. If we were considering asymptomatic individuals and/or multiple external infections then this might not be true. By numerically solving the dynamics we can then calculate the cumulative distribution function (cdf) of the serial interval, 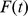, by computing how much probability has flowed into the absorbing states by a given time. We then condition on the index case having created at least one secondary infection before recovering. The solution of the forward equation giving the probability of being in a given state at time 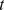 is
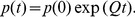
(4)


Removing parts of the state space which are unreachable due to the new absorbing states can reduce the dimension of the matrix and speed up the evaluation of the matrix exponential. The cdf of the serial interval is then given by,
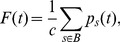
(5)where 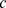 is the probability that the index case infects at least one individual before recovering; note 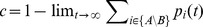. The probability 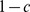 can be calculated simply by considering the sequences of events that would result in the individual going through 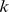 stages without infecting anybody. This then gives,
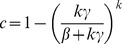
(6)


The serial interval probability mass function is formed by binning into days, as detailed in the next section.

### Likelihood and MCMC algorithm

Given that we can compute the serial interval distribution for a given set of parameters to an arbitrary precision, calculating the likelihood for a given set of serial interval observations is relatively straightforward. Data on the serial interval is generally available at a daily resolution so we always work with a probability mass function binned into days. We used the following binning to calculate the probability of observing a secondary case on the 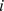 th day [4],
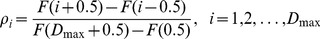
(7)where 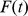 is the cdf and 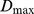 is the maximum range of observations. Given a set of index-secondary case observations, the likelihood of observing them is multinomial with probabilities 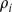. If we have a number of household sizes then the likelihood is just the product of the likelihoods for each household size. MATLAB code to implement this procedure is provided via the Epistruct project [27].

The computational costs of calculating the likelihood are important. The dominant factor is the cost of evaluating the matrix exponential. The number of household sizes has the largest affect on the cost, and also larger households being relatively more expensive than smaller households due to their larger state spaces. Table 2 gives some average times to calculate the likelihood for individual household sizes using a 2.5 GHz Intel core i5 machine running MATLAB. The total average time to calculate the likelihood over 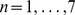 is 0.17 s using the same machine. The number of bins and the overall length of the distribution (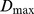) only have small effects on these timings as the EXPOKIT algorithm uses a variable step size [25]. The number of observations has no effect on the computational cost as these enter via a simple multinomial expression.

**Table 2:** Computational costs.

Household size, n	Time (s)	Effective Size
2	0.006	21
4	0.023	48
7	0.045	147

**Figure 1:**
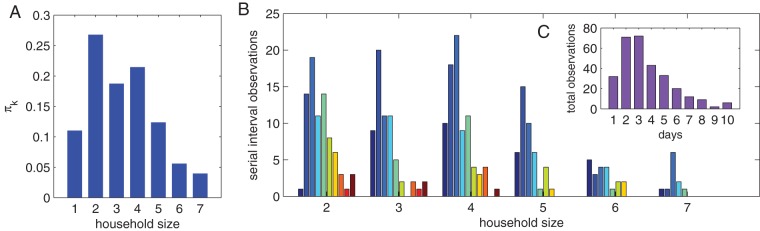
Generated serial interval distributions. (A) shows the size-biased distribution derived from USA 2011 census data. (B) shows 300 randomly generated serial interval observations, stratified by household size. (C) shows the same observations, but summed over all household sizes. These distributions are used in the next section to test the parameter inference methods. Parameters: 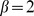, 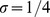, 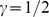 and 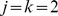.

Average time taken to compute the likelihood for a household of size 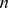. Other parameters 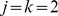, 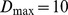 and as given in Figure 1. A 2.5 GHz Intel core i5 machine running MATLAB was used for these timings. The Effective Size is the dimension of the Q matrix once the redundant states have been removed.

The method of inference was Bayesian MCMC [28]. To obtain samples from the posterior distribution we used a Metropolis-Hastings algorithm with independent (truncated) Gaussian proposal densities. In all cases we assumed uniform priors on an interval from zero to an upper bound which depends upon the parameter. Burn-in time was 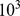 samples and the next 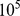 samples were taken, thinned by a factor of 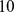 to give 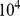 samples from the posterior; convergence was assessed via trace plots. The priors and trace plots for the individual runs are given in Appendix A of File S1.

### Generating test data

To check the robustness of our method we generate a number of serial interval distributions with known household sizes and fixed parameters. We assume the early stages of an epidemic, so the distribution of infected household sizes will be approximately the size-biased distribution, 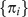, where 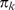 is the probability of a randomly selected individual belonging to a household of size 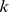 [6], [17]. This is given by
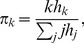
(8)where 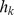 is the household size distribution for a given population. This provides a baseline case, obviously for household clinical trials a different distribution would be appropriate, but in any case it would be explicitly known. Throughout this paper we use census data from U.S.A. 2011 for 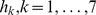, which is shown in figure 1A.

The data is generated by first choosing a random number of household sizes (from 2 to 7) from the size-biased distribution. For each household, a serial interval observation is sampled according to the true distribution binned into days (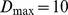). Figure 1B shows the simulated serial interval data stratified by household size. Figure 1C shows the simulated serial interval data summed over all household sizes.

## Results

### Effects of household size

**Figure 2:**
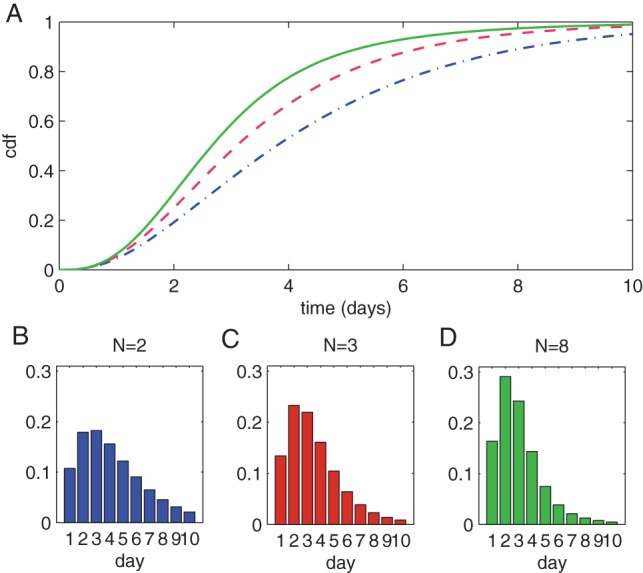
Theoretical serial interval distributions. Part A shows the serial interval cumulative distribution function for households of size 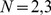 and 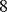 (dot-dashed, dashed and solid lines respectively). Parts B, C and D show the serial interval pmf (binned into days) derived from the corresponding cdfs. Parameter values: 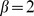, 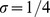, 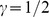 and 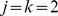.

Figure 2 shows how the serial interval distribution changes with household size, for sizes 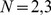 and 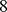 with frequency dependent mixing (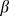 is held constant for different 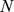). Larger households have higher probability of shorter serial intervals because there are more possibilities for who is the first individual to display symptoms. The change is greatest between 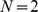 and 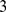, and decreases thereafter. This is because there is a trade-off between more people competing to show symptoms and the fact that these must have been infected later than the first person. As the household size increases the distribution therefore tends to a limiting case. As the variance of the exposed period decreases (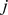 increases) the serial interval also becomes more constant and the difference between the different sized households lessens. The variance of the infectious period (value of 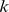) has only a small overall effect on the serial interval distribution, so henceforth we fix 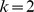 [6], [29], [30].

### Inference with aggregated data

Here we report our findings when attempting to infer the posterior distribution for exposed period parameter 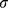 and infectious period parameter 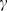 by using the serial interval distribution assuming just a single household size–fixing all other parameters. In later sections we estimate all parameters, but here we are interested in quantifying the biases which can be introduced when using a single household size – effectively ignoring household size – to estimate the serial interval from data which has come from a population consisting of multiple household sizes. This situation often arises when trying to analyse aggregated data from the literature.

**Figure 3:**
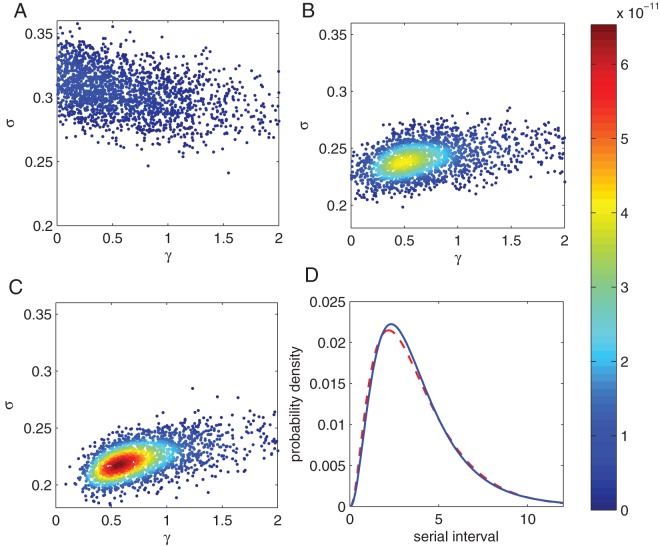
Parameter inference and predicted serial interval distributions. Plots A, B and C show 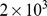 points from the posterior distributions for the parameters 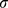 and 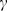 assuming only a single household size, 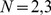 and 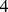 respectively. These are obtained from fitting to the distribution shown in Figure 1C. Points are coloured according to their likelihood with higher values assigned redder shades. All of these introduce a bias in the inferred parameters. Fixed parameters as in Figure 1. Part D shows the mean serial interval distribution for 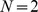 (dashed line) and 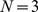 (solid line). The distribution for 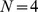 is very close to that for 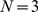 so is not shown. True parameter values: 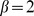, 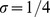 and 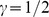.

Figure 3 shows samples from the posterior distributions assuming three different (fixed) household sizes: 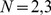 and 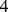. The serial interval data used is that summed over all households, shown in Figure 1C, corresponding to a total of 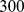 serial interval observations. The case 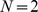 is biased by a large amount away from the true values, severely underestimating the infectious period parameter 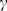 and overestimating the exposed period parameter 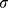. The 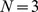 case provides the best estimate of the parameters although there is still bias. Biases arising from using a model with 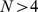 grow larger, with 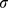 underestimated and 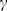 overestimated. The serial interval is most sensitive to the mean exposed period, 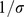, and thus this is more accurately estimated. Although the parameter estimates from the three models are different, the estimated serial interval distributions corresponding to mean parameter estimates are all very similar (see Figure 3D), thus so are the mean serial intervals. The fit using 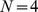 is the best in terms of the mean likelihood.

### Full inference from serial interval observations

**Figure 4:**
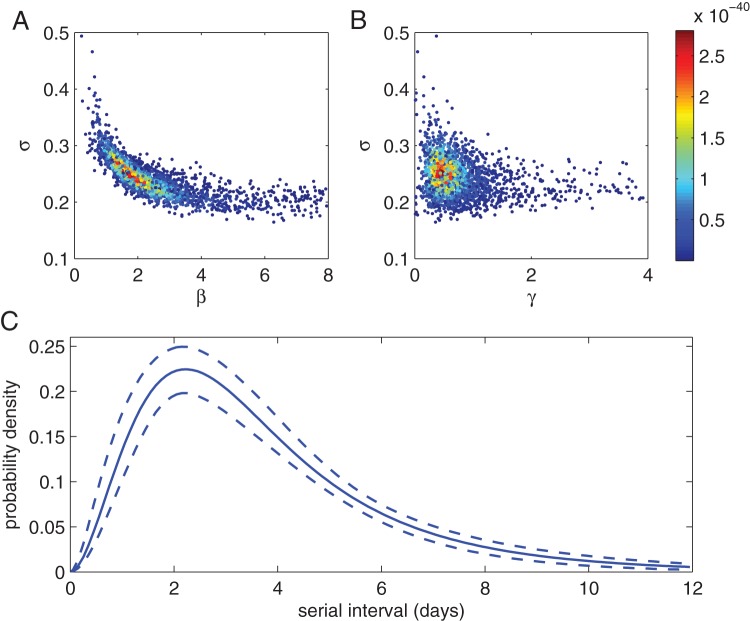
Inference of the serial interval distribution accounting for household size. (A) and (B) show 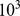 points from the posterior distribution projected along two different parameter axis. Points are coloured according to their likelihood with higher values assigned redder shades. (C) shows the mean serial interval distribution (solid line) and 95% credible intervals (dashed lines) obtained from 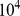 samples of the posterior, summed over all household sizes. True parameter values: 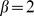, 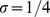 and 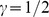.

We now look at estimation of all three variables: transmission parameter 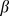, exposed period parameter 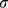 and infectious period parameter 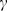, from the generated serial interval observations, given that we also know the household sizes for each observation, i.e. fitting to the data shown in Figure 1B. The variance of the exposed and infectious periods (parameters 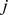 and 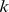) were held fixed. These can be inferred as well, but as noted earlier 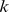 cannot be inferred easily because the serial interval distribution is not very sensitive to it. In contrast the serial interval distribution is typically very sensitive to the variance in the exposed period (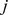) so in practice the actual value is almost always recovered. Figure 4 shows the posterior distributions along with the mean serial interval distribution with credible intervals. The MCMC algorithm for the full inference is appreciably slower than when using just a single household-size model, due to the higher dimension of the search space and need to calculate six individual serial interval distributions for each proposal.

To check the validity of our results we carried out sensitivity analysis. Specifically, we are interested in how the estimated posterior distribution depends on the number of observations available and how it can be skewed due to the random nature of the observations. To assess this we fit the full model to 8 sets of randomly generated serial interval distributions with 15, 50, 100, 200 and 300 data points respectively. The resulting posterior distributions are shown in Appendix B of File S1. The results of this show that exposed period parameter 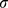 is found accurately most of the time, even for very small sample sizes. The other two parameters, transmission parameter 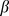 and infectious period parameter 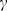 cannot be accurately determined until there are many more samples (typically at least 200). It is likely that we would need to include more of the later infection events within a household to resolve these parameters with more accuracy for smaller sample sizes.

It is also of interest to see how the estimates of parameters can be improved if one of the parameters is already established. We tested this by fixing the transmission parameter, 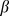, and found the posterior distribution for the other two parameters (figures shown in Appendix C of File S1). This gives an improvement on the posterior for 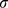, but little improvement for 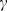. The serial interval distribution derived from this posterior has very similar credible intervals to that shown in Figure 4, so does not give an improved estimate for the mean serial interval.

### Influenza in Hong Kong transmission study

We now use our model to estimate model parameters from a household study in Hong Kong [13]. In this study a Weibull model was fitted to clinical serial interval data corresponding to inter-pandemic influenza in Hong Kong during 2007. This was then used to estimate the mean serial interval with parametric bootstrapping to calculate confidence intervals [13]. Admittedly this has a very small sample size (only 14 observations from households of sizes 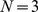 to 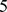), but serves to illustrate the power of our method with real data. It is also the only study we have found which explicitly gives household size with serial interval observations. In the original study it was shown that external rates of infection had no impact on the serial interval estimate, so our model is appropriate to analyse this data.

To investigate the sensitivity to the variance of the exposed period we separately fitted two versions of the model with 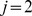 and 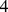. The higher value of 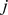 gives a more constant exposed period. As in the previous section we estimate the three parameters 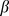, 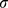 and 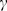 and set 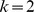. Full details of the MCMC routine are given in Appendix A of File S1. Only the posterior for the exposed period parameter, 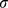, could be determined to within reasonable limits. Both values of 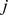 gave similar results: for 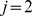, 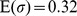 (95% CI 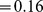
–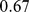), and for 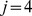, 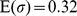 (95% CI 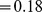
–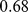). The distributions for both the transmission parameter 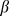 and infectious period parameter 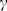 were not well determined, but this is expected given the results of the sensitivity analysis in the previous section.

**Figure 5:**
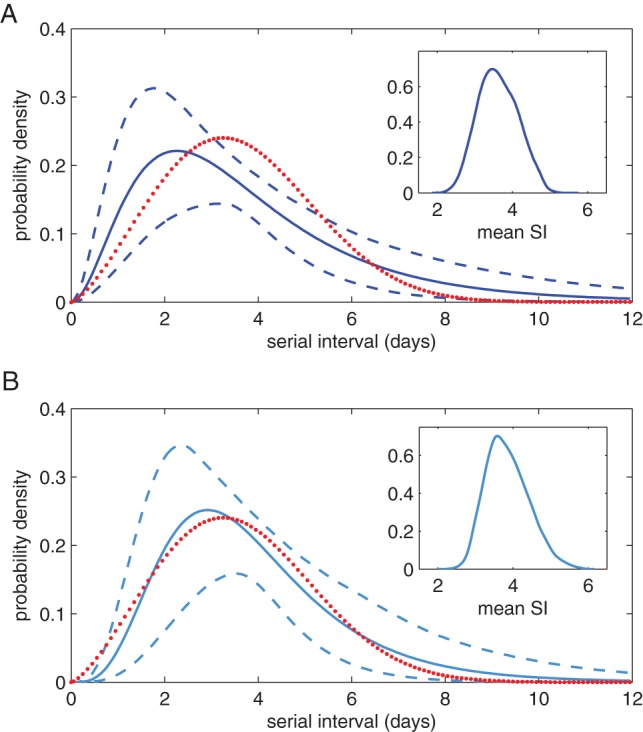
Estimated serial interval distributions. Solid lines depicts the mean and dashed lines the 95% credible intervals assuming 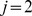 (A) and 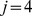 (B). The dotted lines show the Weibull distribution estimated in the original analysis [13]. The insets show kernel density plots for the mean serial interval.

**Figure 6:**
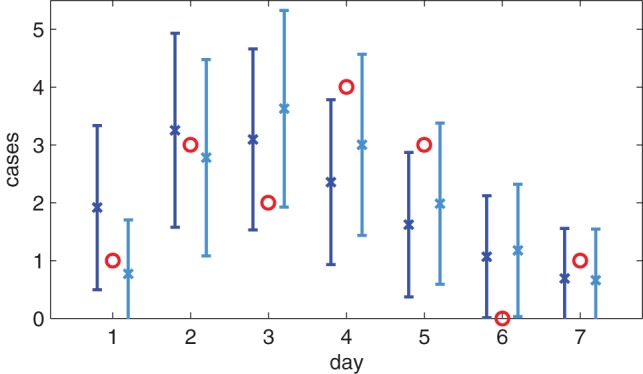
Expected number of serial interval observations of each duration compared with original data. Original data is summed over household size shown as circles (14 observations in total). The markers with bars show the mean and one standard deviations for the 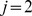 fit (x markers) and the 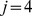 fit (square markers).

The estimated serial interval distributions and credible intervals are shown in Figure 5 for the two different values of 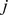, along with kernel density plots for the mean serial intervals. In the original analysis a Weibull distribution was fitted [13] and is shown for comparison; the estimated mean serial interval was 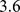 days (95% confidence interval 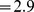
–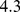). From the serial interval distributions we estimate the mean serial interval to be 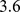 days (95% CI 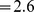
–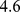) assuming 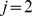 and 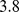 days (95% CI 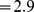
–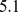) assuming 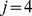. The mean likelihood of the 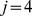 fit is approximately three times that of the 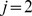 fit. Figure 6 shows the expected number of serial interval observations of each duration and standard deviations for the two fits compared to the original data. For the 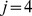 case all the data lies within one standard deviation.

## Discussion

The serial interval is relatively easy to observe and has been shown to be critically important for predicting disease dynamics and choosing control policies. For these reasons it is commonly recorded during the early stages of a pandemic. The difficulty arises when attempting to use the observations for modelling, or inference, because the serial interval is the convolution of two processes: infection and incubation, and the infection time is effectively unobservable.

In this paper we have provided methodology for parametrising a quite general Markovian model of household disease dynamics to serial interval data. Not only does this approach provide an estimate of the distribution of serial interval, but it also provides an estimate of a mechanistic model of the disease dynamics. This approach facilitates the prediction of disease dynamics and the assessment of alternative control options, of much importance in the early stages of disease invasion.

We have shown how the distribution of serial interval can be evaluated to arbitrary precision for our stochastic households model. Unlike stochastic simulation, which is computationally intensive and produces an estimate, our method is efficient and allows precise likelihood evaluation. Analytical results can be derived, using approximations in the cases 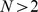 (see Appendix D of File S1), but in practice these offer no advantage over the numerical scheme due to the unwieldy nature of the expressions derived.

Our model allows us to quantify the effect of household size on the clinical serial interval (the time between first and second showing of symptoms, assuming that there is no asymptomatic infection and only a single introduction), hence identifying its importance for estimation. By fitting to serial interval distributions stratified by household size we can can obtain accurate posterior distributions for all three of the basic parameters: transmission parameter 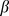, exposed period parameter 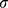, and infectious period parameter 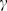. The parameter 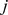, controlling the variance of the exposed period, can also be inferred, although we have not implemented this within the MCMC scheme. The serial interval distribution is relatively insensitive to the parameter 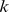, controlling the variance in the infectious period, so we have not attempted to infer this and have held it constant. If full time series of `symptomatic' events are available then our method is potentially wasteful because we do not use the later events. Our methods can be extended to inference of full time series and it is likely that this is required to get better estimates on the parameters 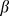 and 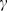. Such a project is currently under way.

We have shown the effectiveness of this scheme for estimating parameters from simulated data as well as data from a Hong Kong influenza study [13]. Despite the small sample size we could still infer meaningful estimates for the exposed period and serial interval distribution, consistent with the earlier study. This demonstrates that the methodology reliably produces estimates as would be obtained via traditional parametric fitting, but has the added benefit of producing estimates of parameters for our stochastic, mechanistic model of disease dynamics. Of course, one must be careful in using household quantities to make population level predictions [9]; to do this we typically need to make more assumptions about population level mixing and transmission. In related work on antiviral effectiveness [6] we have used this method with a simpler model to effectively estimate the exposed period parameter 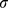 and infectious period parameter 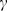 from a larger influenza serial interval dataset [4]. Although this dataset was larger, the data was not stratified by household size, so we had to use a mean household size in our estimation. This then allowed us to evaluate posterior distributions for population level quantities such as the household basic reproductive number, 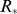, and early growth rate [17], [21], [31].

The serial interval is also important because of its relation to the generation time which can be used to relate the Malthusian early growth rate, 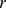, and the basic reproductive ratio, 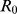 [8], [9], [14], [23]. Usually it is assumed that these two distributions have the same mean, but in general their distributions will be different [32]. The actual generation time distribution can be derived for our model in a similar way to the serial interval distribution. Briefly, one would change the initial condition of the Markov chain to 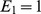 and make a different set of states absorbing. Once the joint posterior distribution for the parameters is inferred from the serial interval data, we can use it to compute the generation time distribution.

Whilst our model is quite general, there exists a number of features which may be required for particular diseases, populations and data sets which would require modification of our approach. For example, we have not explicitly accounted for external infection and co-primary cases, varying infectiousness with stage of infection, or symptoms that do not coincide with the commencement of infectiousness. It possible to extend the model to account for these features, and the method we have outlined will also need to be modified slightly to accommodate these extensions. We note that in all cases extra parameters will require estimation. We are currently developing and testing such frameworks. However, the model we have explicitly analysed herein is of much interest in infectious disease modelling, and the method we have detailed will facilitate its use in the early stages of disease invasion, of much interest to public health policy.

Here we have shown that household size has a significant impact on the serial interval, and that including this data improves estimates. Throughout we have assumed frequency-dependent transmission, as appears to be most appropriate for influenza in households [15], but one would expect the differences to be exacerbated by density-dependent transmission – not only do larger households have more individuals competing to display symptoms first, but the transmission rate would also be larger for the same household configuration which further reduces the serial interval. Household size is typically recorded alongside the serial interval, so our method simply proposes a way to make appropriate use of this routinely collected data; an approach which has the benefit of producing posterior distributions of parameters corresponding to a fully-mechanistic model of the disease dynamics.

## Supporting Information

File S1This file contains Statistical (MCMC) details; Sensitivity analysis of the full model; Full inference while holding 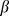 fixed; and, Some analytic results.(PDF)
