# Supplementary material for: Estimating a Markovian Epidemic Model Using Household Serial Interval Data from the Early Phase of an Epidemic
Source: PLoS One. 2013 Aug 30;8(8):e73420. doi: 10.1371/journal.pone.0073420 (PMC3758268; doi:10.1371/journal.pone.0073420)
Supplement: File S1 — This file contains Statistical (MCMC) details; Sensitivity analysis of the full model; Full inference while holding fixed; and, Some analytic results. (PDF) [file pone.0073420.s001.pdf]

Estimating a Markovian epidemic model using household serial interval data from the early phase of an epidemic: Supplementary materials

Andrew J. Black[1] and Joshua V. Ross[1]

June 3, 2013

1. School of Mathematical Sciences, The University of Adelaide, Adelaide SA 5005, Australia.

A Statistical (MCMC) details

Here we give details of the MCMC routines used in the results sections. In all cases the proposals were Gaussian. Burn-in was 10^3 samples and the next 10^5 samples were taken, thinned by a factor of 10 to give 10^4 samples from the posterior. Table 1 gives the parameters for the full inference model. Table 2 gives the parameters used in the Hong Kong influenza inference.

**Parameter**	**True value**	**Prior**	**Sampling variance**
β	2	$U(0,8)$	0.05
σ	0.25	$U(0,2)$	0.01
γ	0.5	$U(0,4)$	0.02

Table S1: MCMC parameters for the full inference model.

**Parameter**	**Prior**	**Sampling variance**
β	$U(0,2)$	0.05
σ	$U(0,1)$	0.01
γ	$U(0,2)$	0.02

Table S2: MCMC parameters for the Hong Kong model.

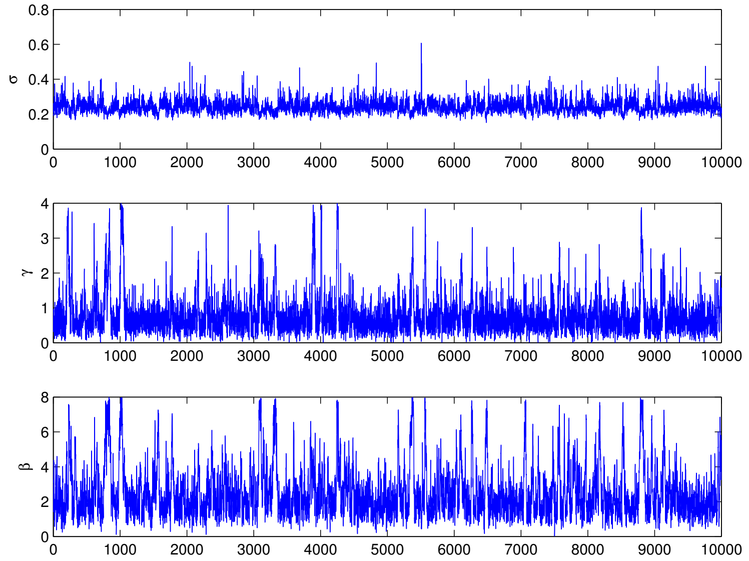

Figure S1: Post thinning trace plots for the full inference model

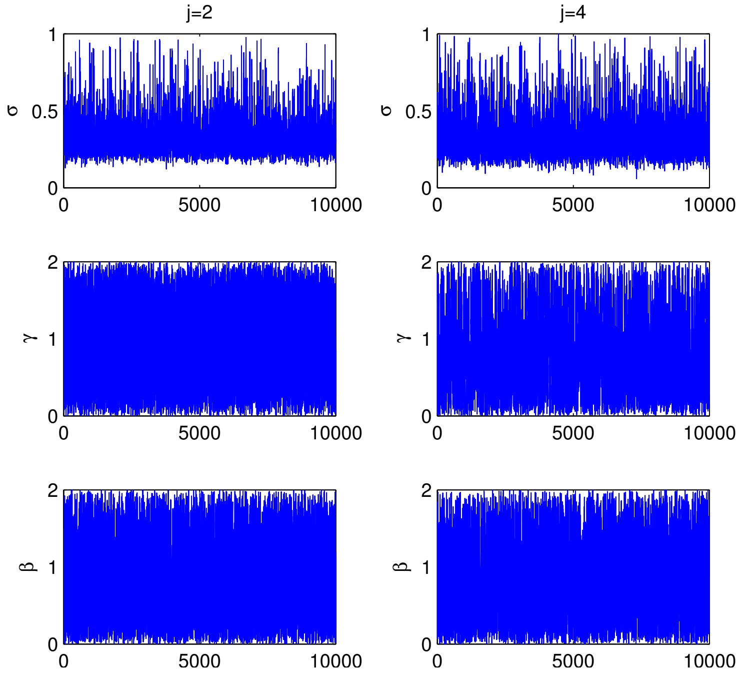

Figure S2: Post thinning trace plots for the two fits to the Hong Kong influenza data set.

B Sensitivity analysis of the full model

Here we show the results from a sensitivity analysis. We are mostly concerned with how the number of data points and their random nature affect the posterior distribution. Figures 3-7 show the posterior distributions for 8 sets of randomly generated serial interval distributions with 15, 50, 100, 200 and 300 data points respectively. The model parameters are: $\beta = 2$, $\sigma = 1/4$, $\gamma = 1/2$ and $j = k = 2$. MCMC parameters are given in Table 1.

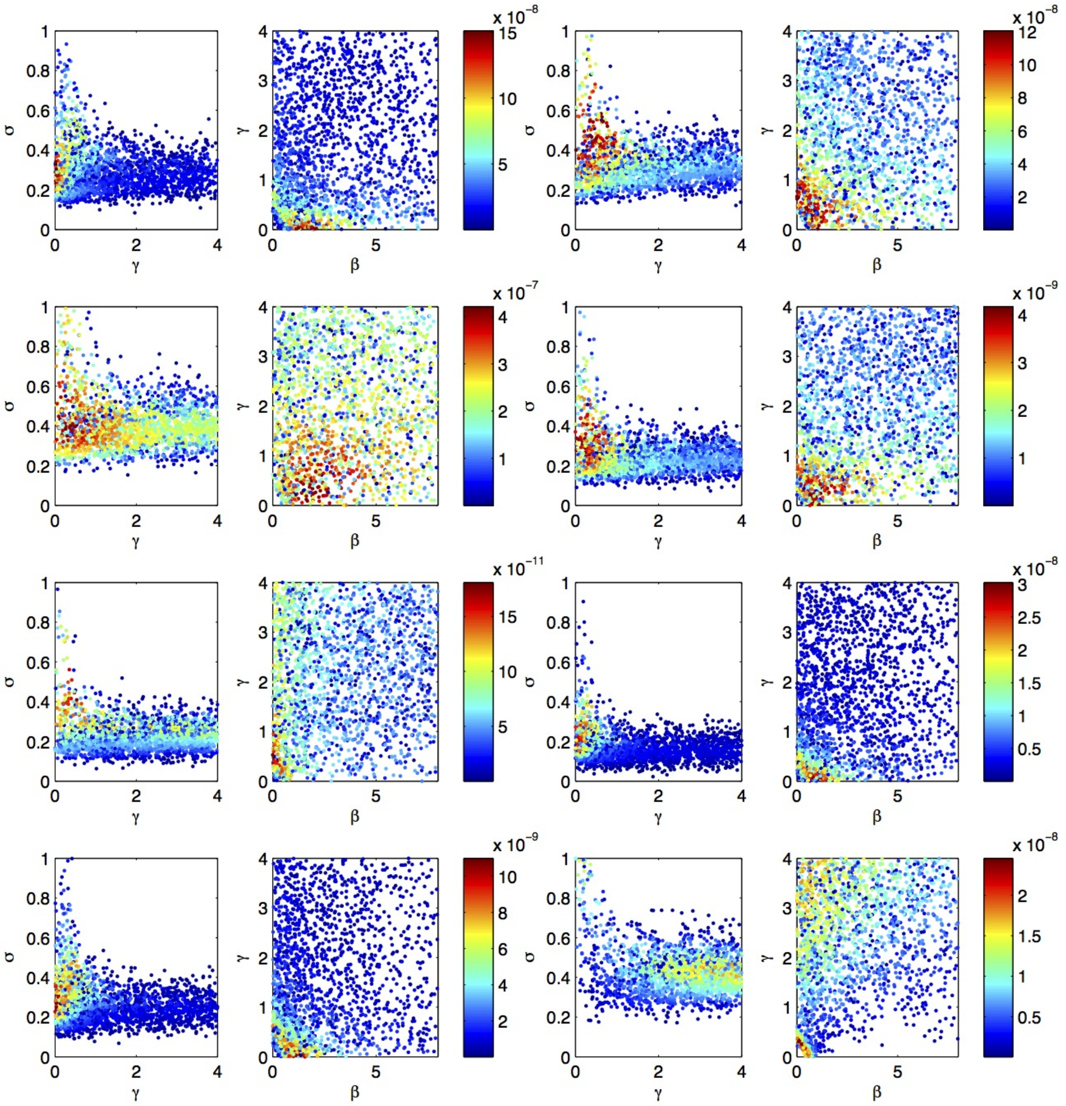

Figure S3: Posterior distributions from the full model for 8 serial interval distributions comprised of 15 random data points. Points are coloured according to their likelihood values.

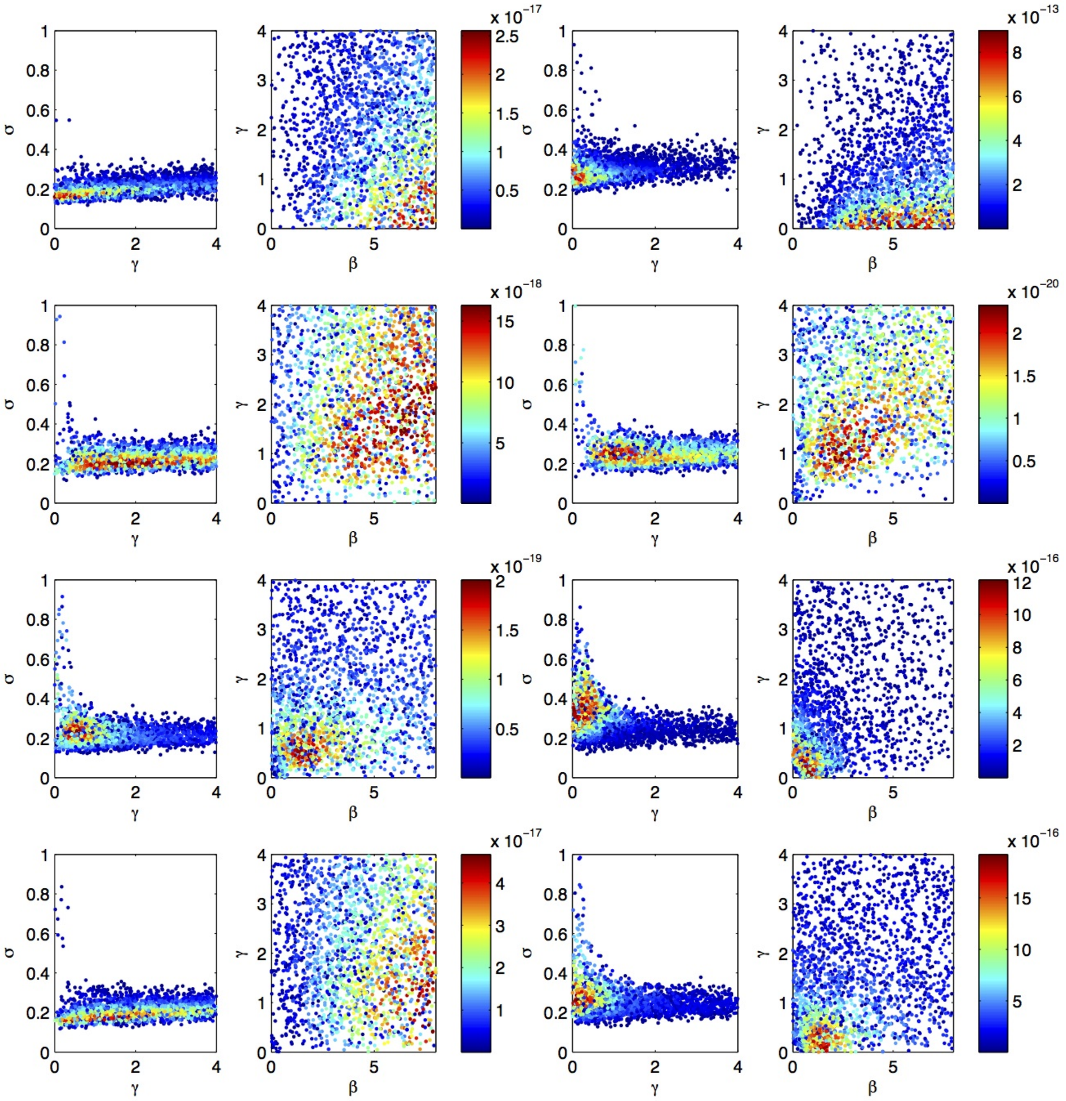

Figure S4: Posterior distributions from the full model for 8 serial interval distributions comprised of 50 random data points. Points are coloured according to their likelihood values.

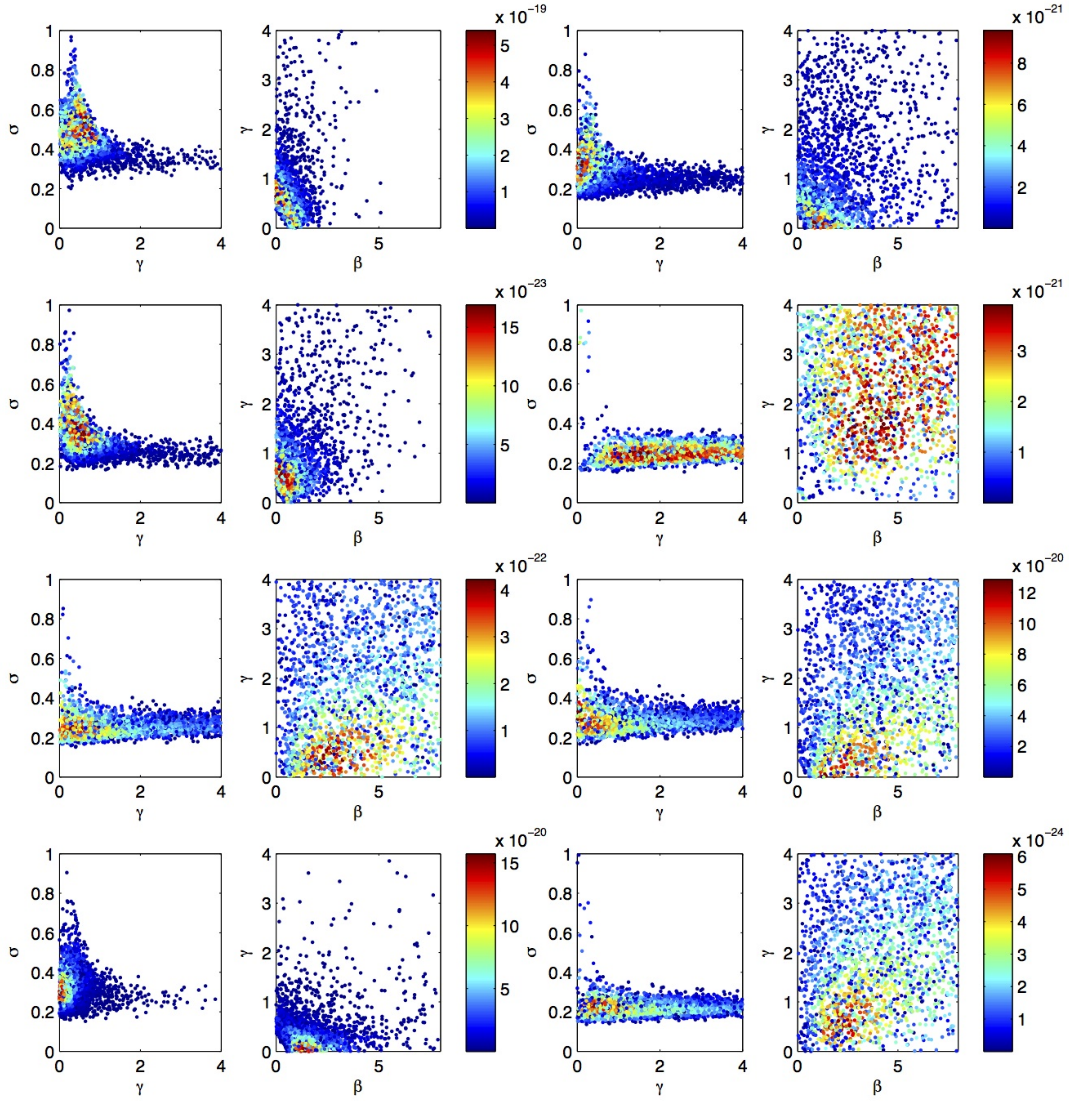

Figure S5: Posterior distributions from the full model for 8 serial interval distributions comprised of 100 random data points. Points are coloured according to their likelihood values.

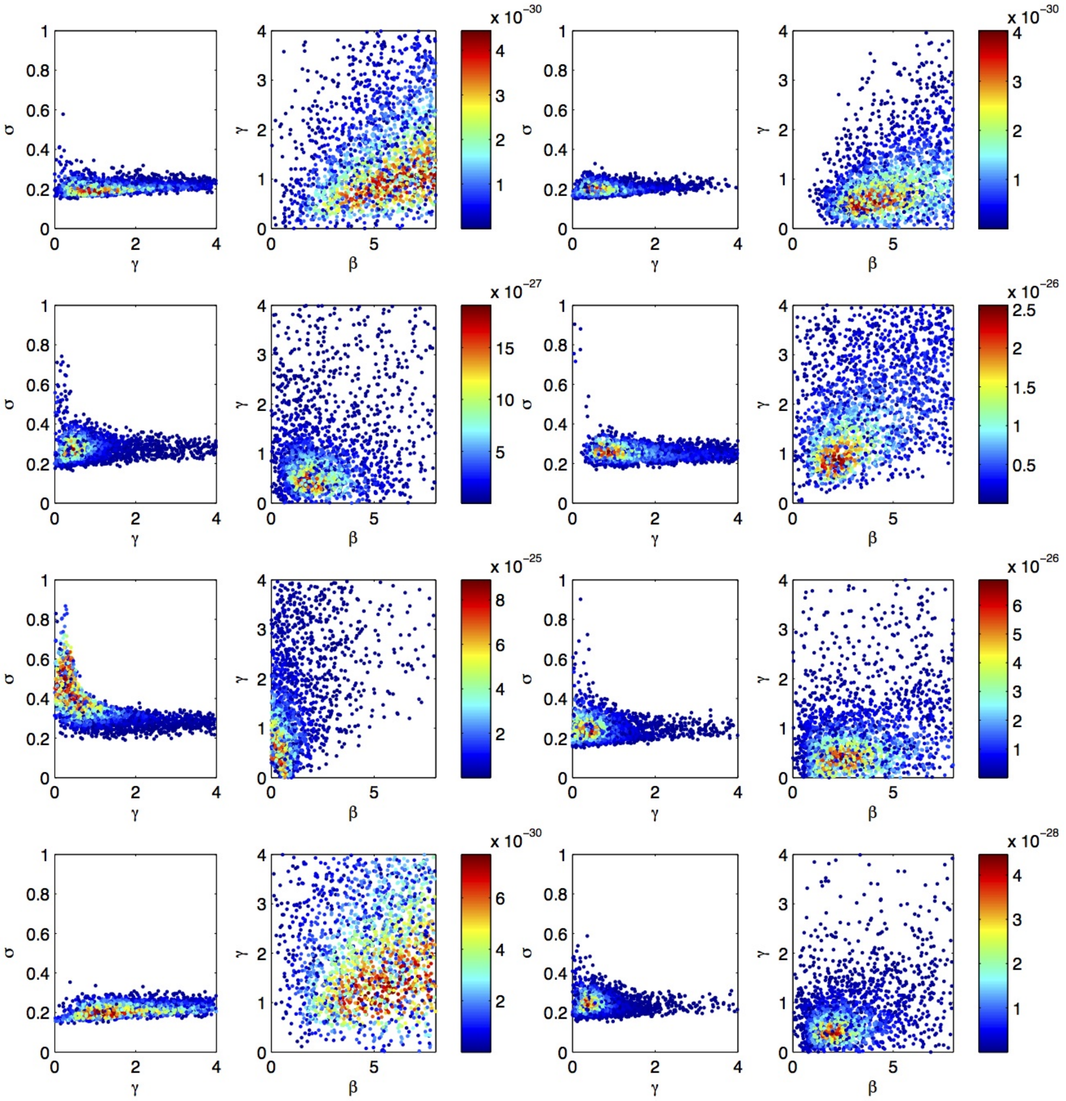

Figure S6: Posterior distributions from the full model for 8 serial interval distributions comprised of 200 random data points. Points are coloured according to their likelihood values.

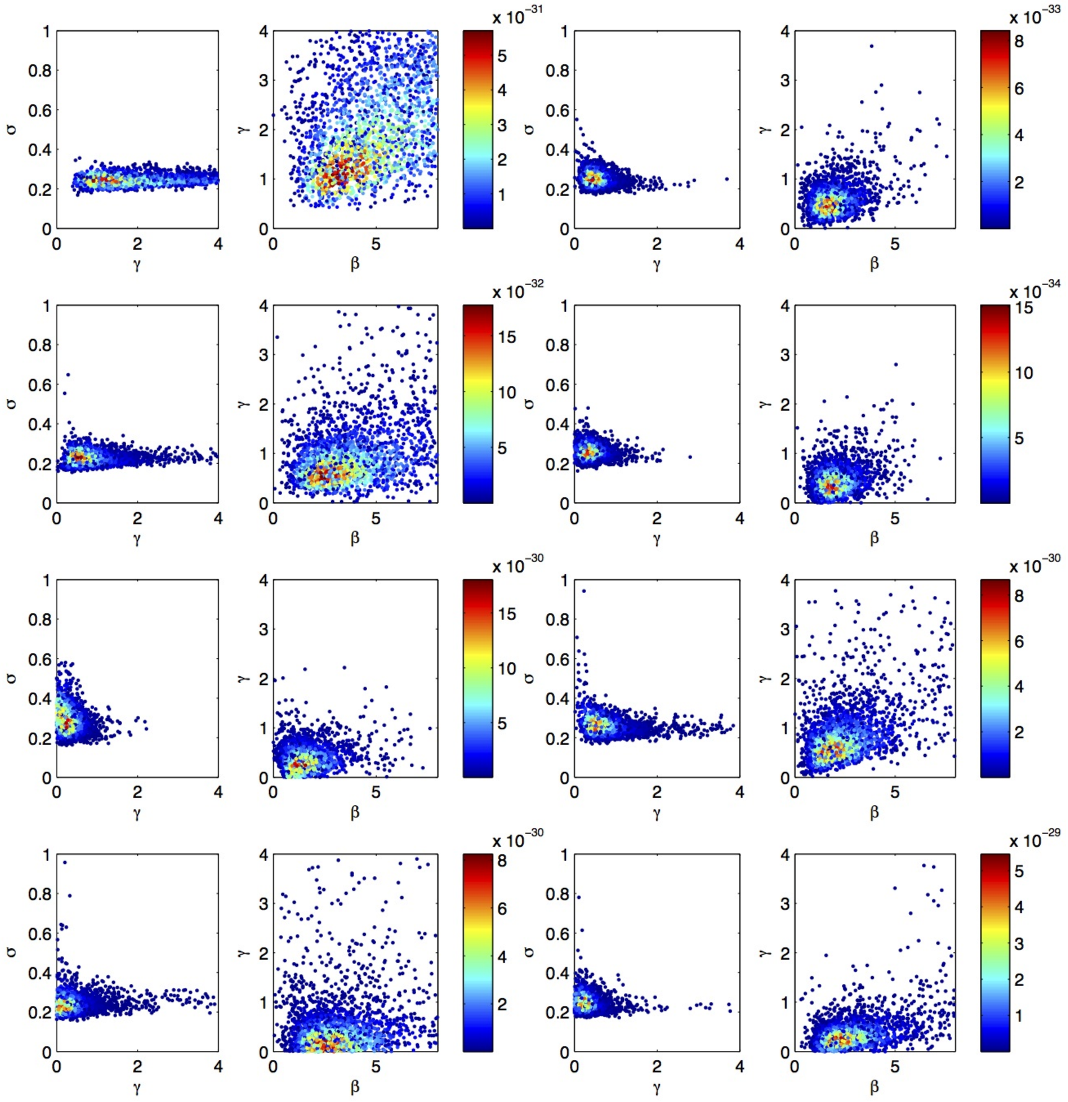

Figure S7: Posterior distributions from the full model for 8 serial interval distributions comprised of 300 random data points. Points are coloured according to their likelihood values.

C Full inference while holding β fixed

Here we present results from fitting the full model when the parameter β is fixed. Figure S8 shows posteriors estimated from 9 random serial interval distributions. Figure S9 shows the mean serial interval distribution and confidence intervals computed from fitting to the full distribution shown in the main text and holding β fixed.

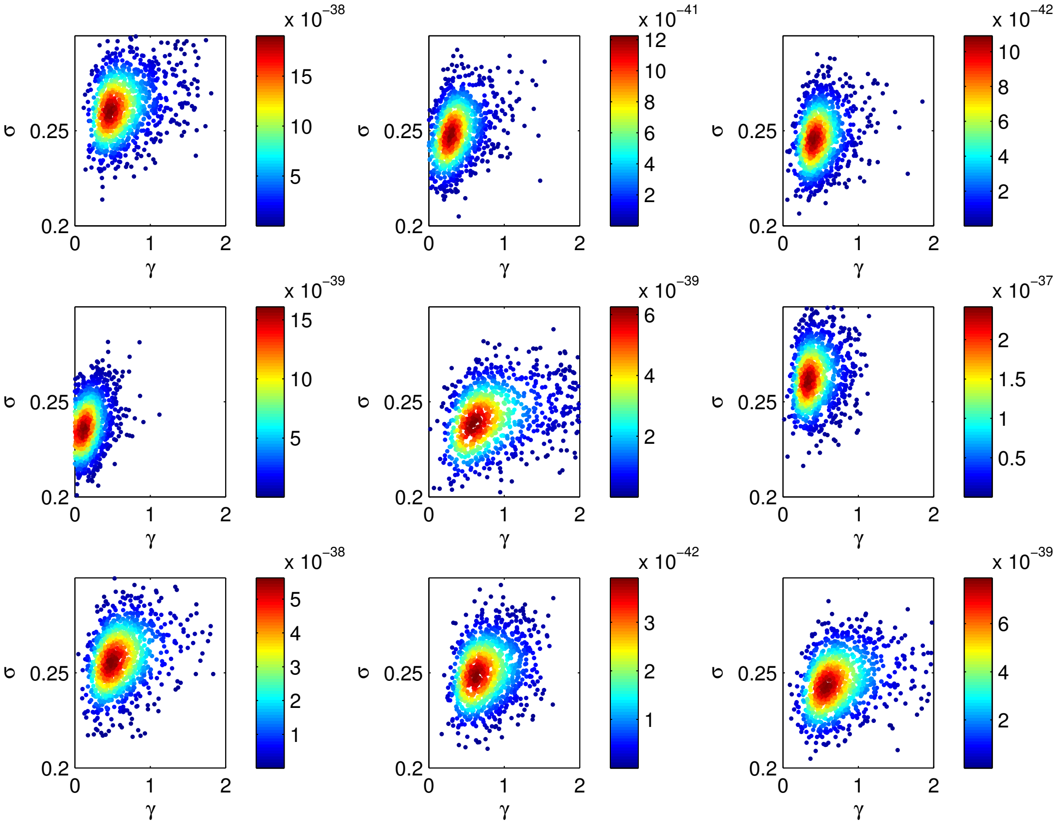

Figure S8: Estimating parameters using full household size distribution. Each plot shows 10^3 samples from the posterior, estimated from 300 random observations of the serial interval in households of size 2 to 7. Fixed parameters: $\beta = 2$, $j = k = 2$.

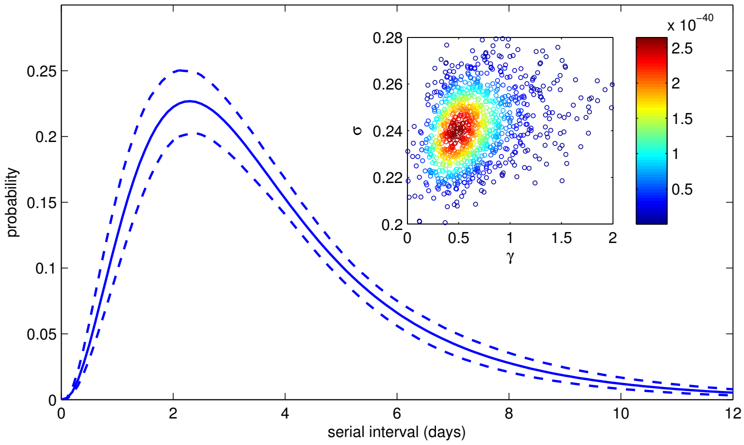

Figure S9: Inference of the serial interval distribution accounting for household size. Inset shows 10^3 points from the posterior distribution when accounting for household size, i.e. fitting to the full distribution shown in Figure 1B of the main text. Points are coloured according to their likelihood. The main graph shows the mean serial interval distribution and 95% credible intervals obtained from 10^4 samples of the posterior, summed over all household sizes.

D Some analytic results

The case $N = 2$*.* The cdf of the clinical serial interval T_{SI} in the case $N = 2$ is given by

$$\begin{aligned} \Pr(T_{SI} \le t) &= \Pr(T_{Inf} + T_{Sym} \le t) \\ &= \int_0^t \Pr(T_{Sym} \le s) \Pr(T_{Inf} \le t - s) ds \\ &= \int_0^t f_{T_{Sym}}(y_1) \sum_{m=1}^{k} \rho(m) \prod_{i=1}^{m} \int_0^{t - \sum_{l=1}^{i} y_l} f_{T_{Inf,i}}(y_{i+1}) dy_1 dy_{i+1}, \end{aligned} \tag{1}$$

where T_{Inf} is the time for the first individual to infect the second individual, and T_{Sym} is the time for the second individual to display symptoms. The exposed period is distributed $\Gamma(j, j\sigma)$, and so the probability density function

$$f_{T_{Sym}}(x) = \frac{j\sigma e^{-j\sigma x}(j\sigma x)^{j-1}}{(j-1)!}.$$

Now, when considering the random variable T_{Inf}, we have k different possible paths to the occurrence of the first secondary case, corresponding to the infectious class of the index case at time of transmission. Each of these paths has a corresponding probability $\rho(m)$ of arising (with $\sum_{m=1}^{k} \rho(m) = 1$, as a secondary case is assumed to

occur). These probabilities may be evaluated by calculating the probability of the specified sequence of events, via the jump chain of the Markov chain conditioned on a secondary case. The corresponding holding times in each state of the conditioned chain are exponentially distributed, with some rate λ_i. Hence,

$$f_{T_{Inf,i}}(y_{i+1}) = \lambda_i e^{-\lambda_i y_{i+1}}.$$

We now consider the evaluation of the required probabilities conditioned on a secondary case, in order to evaluate $\rho(m)$ and the rates λ_i. To evaluate these quantities, we use the results of Waugh [1] on *conditioned Markov processes*. The transition rates of the conditioned process are

$$\tilde{q}_{ij} = \begin{cases} \frac{p_j}{p_i} q_{ij}, & i \in F \\ q_{ij}, & i \notin F, \end{cases}$$

where F is the set of states from which the event of interest, the first secondary case, can occur, and p_i is the probability of the event of interest, at least one secondary case, occurring starting from state i. For the model with k infectious classes, the probability of at least one secondary case from state i is

$$p_i = \begin{cases} \alpha_{k-n} := \left(\frac{\beta}{\beta+k\gamma}\right) \sum_{m=0}^{n} \left(\frac{k\gamma}{\beta+k\gamma}\right)^m, & i_1 = N-1, i_{j+k-n} = 1 \\ 0, & \textit{otherwise}, \end{cases}$$

for $0 \leq n \leq k$, and where state $i = (i_1, i_2, \ldots, i_{j+1}, i_{j+2}, \ldots, i_{j+k}) = (N-1, 0, \ldots, 0, v_{k,1})$. Now, finally, the rates

$$\lambda_i = \sum_m \tilde{q}_{i,m}.$$

We note that only two types of events can occur in the conditioned process from the states $(E_1, E_2, \ldots, E_j, I_1, I_2, \ldots, I_k, R) = (0, 0, \ldots, 0, e_{k-1,i}, 0, 0)$, where $e_{k-1,i}$ is a vector of length $k-1$ with a 1 in the ith position, and they are to states $(0, 0, \ldots, 0, e_{k,i+1}, 0)$ (corresponding to *Progression* of infectious class) and to $(1, 0, \ldots, 0, e_{k-1,i}, 0, 0)$ (corresponding to a new *Infection*); hence we denote the rates of these events, for notational convenience, by $\tilde{q}_{i,P}$ and $\tilde{q}_{i,I}$ respectively, and are given by

$$\tilde{q}_{i,P} = \left(\frac{\alpha_{i+1}}{\alpha_i}\right) k\gamma$$

and

$$\tilde{q}_{i,I} = \left(\frac{1}{\alpha_i}\right) \beta.$$

In state $(0, 0, \ldots, 0, 0, \ldots, 1, 0)$ the only transition possible, in the conditioned process, is a transition to state $(1, 0, \ldots, 0, 0, \ldots, 1, 0)$ at rate $q_k = \beta/\alpha_k$.

Hence, the probability the first infection occurs with the index case in stage $m(< k)$ of the k stages, in the conditioned process, is given by

$$\begin{aligned}\rho(m) &= \left(\frac{\left(\frac{1}{\alpha_m}\right)\beta}{\left(\frac{1}{\alpha_m}\right)\beta + \left(\frac{\alpha_{m+1}}{\alpha_m}\right)k\gamma}\right)\left(\prod_{i=1}^{m-1}\frac{\left(\frac{\alpha_{i+1}}{\alpha_i}\right)k\gamma}{\left(\frac{1}{\alpha_i}\right)\beta + \left(\frac{\alpha_{i+1}}{\alpha_i}\right)k\gamma}\right)\\ &= \left(\frac{\beta}{\beta + \alpha_{m+1}k\gamma}\right)\left(\prod_{i=1}^{m-1}\frac{\alpha_{i+1}k\gamma}{\beta + \alpha_{i+1}k\gamma}\right)\end{aligned}$$

with an empty product evaluating to one, and

$$\rho(k) = \left(\prod_{i=1}^{k-1}\frac{\alpha_{i+1}k\gamma}{\beta + \alpha_{i+1}k\gamma}\right).$$

Finally, note that

$$\lambda_i = q_{i,I} + q_{i,P} = \frac{\beta + \alpha_{i+1}k\gamma}{\alpha_i}, \quad i < k$$

and $\lambda_k = q_k = \beta/\alpha_k$.

Substituting these equations into (4), it is possible to evaluate the cdf of the CSI in the case $N = 2$ explicitly, with the assistance of MAPLE, at least for fixed j and k.

The $N > 2$ case. Unfortunately, the cdf of CSI in the case $N > 2$ is more challenging to evaluate than the $N = 2$ case. We now have

$$\begin{aligned}\Pr(T_{SI} \le t) &= \Pr\left[(T^1_{Inf} + T^1_{Sym} \le t) \cup (T^1_{Inf} + T^2_{Inf} + T^2_{Sym} \le t)\ldots\right]\\ &= \Pr\left[\bigcup_{i=1}^{N-1}\left(T^i_{Sym} + \sum_{j=1}^{i} T^j_{Inf} \le t\right)\right]\\ &= \Pr\left[\bigcup_{i=1}^{N-1}(A_i \le t)\right]\\ &= \sum_{i=1}^{N-1}\Pr(i \text{ secondary infections})\Pr(\min\{A_1,\ldots,A_i\} \le t).\end{aligned}$$

The difficulty in evaluating this arises due to the dependence of the $T^1_{Inf},\ldots,T^{i-1}_{Inf}$ random variables within A_{i-1} and A_i. For this reason, we consider an approximation which is derived by assuming independence between these random variables.

We therefore have

$$\begin{aligned}\Pr(T_{SI} \le t) &= \sum_{i=1}^{N-1}\Pr(i \text{ secondary infections})\Pr[\min\{A_1,\ldots,A_i\} \le t]\\ &= \sum_{i=1}^{N-1}\Pr(i \text{ secondary infections})\left[1 - \prod_{j=1}^{i}\Pr(A_j > t)\right]. \qquad (2)\end{aligned}$$

We can evaluate the probabilities $\Pr(A_j > t)$ using our results presented in the previous section, for evaluating the distribution of the CSI in the $N = 2$ case. We simply need to account for the probability of starting in each infectious class for the jth infection, and modify the transmission rate taking into account past infections. Due to the algebraic complexity of the resulting expressions, even under the assumption of independence, we once again use MAPLE for evaluation purposes. This also comes with the benefit that we may easily exploit Laplace transforms of pdfs in order to evaluate the pdf of each A_j ($j > 1$), via the product of the Laplace transforms of the individual pdfs which follows from our assumption of independence. Finally, due to the small number of individuals which constitute households, we can evaluate the probability of having i secondary infections, for $1 \leq i \leq N - 1$ (conditioned on at least one secondary infection).

Unfortunately, the resulting expressions are so lengthy that the are of limited use. From a practical viewpoint, they take longer to evaluate than the numerical scheme outlined and implemented in the paper. Whilst the approximation is typically accurate, the fact that it is an approximation and combined with this computational inferiority, we have chosen to use only the numerical method herein.

References

[1] W Waugh. Conditioned markov processes. *Biometrika*, 45:241–249, 1958.
